# Virtual reality against Zoom fatigue? A field study on the teaching and learning experience in interactive video and VR conferencing

**DOI:** 10.3205/zma001601

**Published:** 2023-04-17

**Authors:** Robert Speidel, Edward Felder, Achim Schneider, Wolfgang Öchsner

**Affiliations:** 1 Ulm University, Medical Faculty, Division of Learning and Teaching, Competence Center eEducation in Medicine, Ulm, Germany; 2 Ulm University, Institute of General Physiology, Ulm, Germany; 3 Ulm University, Faculty of Medicine, Division of Learning and Teaching, Ulm, Germany; 4 University Hospital Ulm, Clinic for Anesthesiology and Intensive-Care Medicine, Ulm, Germany

**Keywords:** virtual reality, social VR, distance learning, Zoom fatigue, medical education

## Abstract

**Aim:**

During the COVID-19 pandemic, the absence of in-person teaching was partially compensated for through videoconferencing. However, lecturers complain that students do not participate actively in video-based online seminars. One reason cited for this is Zoom fatigue. Conferences in virtual reality (VR), accessible with and without head-mounted display, represent one potential remedy to this issue. The research to date does not shed any light on the (1.) teaching experience, (2.) student demand, (3.) learning experience (including participation and social presence), and (4.) learning performance (declarative and spatial) associated with VR conferences. The present work will compare these aspects for videoconferencing, independent study, and – in the case of teaching experience – with in-person teaching.

**Methods:**

A compulsory seminar in General Physiology was offered during the 2020/21 winter semester and the 2021 summer semester as part of the Human Medicine program at the Faculty of Medicine at Ulm University. The seminars were offered in three different formats with identical content: (a) VR conference, (b) video conference, and (c) independent study, with students selecting the format of their choice. In the VR conferences, the lecturer taught using a head-mounted display while students participated via PC, laptop, or tablet. The learning experience and learning performance were assessed using questionnaires and a knowledge test. A semi-structured interview was conducted to assess the VR teaching experience.

**Results:**

The lecturer's teaching experience in the VR conferences was similar to in-person teaching. Students predominantly chose independent study and videoconferencing. The latter resulted in worse outcomes with regard to learning experience (including participation and social presence) and spatial learning performance than the VR conferences. Declarative learning performance differed only slightly between teaching formats.

**Conclusions:**

VR conferencing offers lecturers new didactic opportunities and a teaching experience similar to that of in-person teaching. Students prefer time-efficient videoconferencing and independent study, but rate participation and social presence, among other things, higher in VR conferencing. If faculty and students are open to the technology, VR conferencing can promote interactive exchange in online seminars. This subjective assessment is not associated with better declarative learning performance.

## Zusammenfassung

**Zielsetzung:**

Während der Corona-Pandemie wurde der Ausfall der Präsenzlehre teils mit Videokonferenzen kompensiert. Dozierende beklagen jedoch, dass sich Studierende nicht aktiv an videobasierten Online-Seminaren beteiligen. Ein Grund dafür sei Zoom-Fatigue. Eine Abhilfe könnten Konferenzen in der virtuellen Realität (VR) sein, die mit und ohne VR-Brille zugänglich sind. Die bisherige Forschungslage gibt allerdings keinen Aufschluss über die mit VR-Konferenzen verbundene (1.) Lehrerfahrung, (2.) studentische Nachfrage, (3.) Lernerfahrung (u.a. Beteiligung und soziale Präsenz) und (4.) Lernleistung (deklarativ und räumlich). Diese Aspekte sollen im curricularen Einsatz mit Videokonferenzen, dem Selbststudium und – im Falle der Lehrerfahrung – mit dem Präsenzunterricht verglichen werden.

**Methodik:**

Im Studiengang Humanmedizin der Medizinischen Fakultät Ulm wurden im WiSe 2020/21 und SoSe 2021 jeweils ein Pflichtseminar der Allgemeinen Physiologie in drei verschiedenen Lehrformaten inhaltsgleich angeboten: (a) VR-Konferenz, (b) Videokonferenz und (c) Selbststudium. Die Studierenden wählten ihr Lehrformat selbst. In den VR-Konferenzen lehrte der Dozierende mit VR-Brille, während die Studierenden via PC, Laptop oder Tablet teilnahmen. Die Lernerfahrung und die Lernleistung wurden mit Fragebögen und einem Wissenstest untersucht. Für die Lehrerfahrung in VR wurde ein halb-strukturiertes Interview durchgeführt.

**Ergebnisse:**

In den VR-Konferenzen stellte sich beim Dozierenden ein Lehrerlebnis wie in Präsenz ein. Die Studierenden wählten vorwiegend das Selbststudium und die Videokonferenzen. Letztere schnitten bei der Lernerfahrung (u.a. Beteiligung und soziale Präsenz) und der räumlichen Lernleistung schlechter ab als die VR-Konferenzen. Die deklarative Lernleistung unterschied sich nur geringfügig zwischen den Lehrformaten.

**Schlussfolgerungen:**

VR-Konferenzen bieten Dozierenden neue didaktische Möglichkeiten und ein Lehrerlebnis wie in Präsenz. Studierende bevorzugen zeiteffiziente Videokonferenzen und das Selbststudium, bewerten in VR-Konferenzen jedoch u.a. die Beteiligung und soziale Präsenz besser. Wenn Dozierende und Studierende für die Technologie aufgeschlossen sind, können VR-Konferenzen den interaktiven Austausch in Online-Seminaren begünstigen. Diese subjektive Einschätzung geht nicht mit einer besseren deklarativen Lernleistung einher.

## 1. Background

### 1.1. Interaction in videoconferences

Videoconferencing technology allows teaching sessions to be held and attended independently of location. During the COVID-19 pandemic, this partially compensated for the absence of in-person teaching [1]. However, lecturers complain about a lack of student participation in video-based online seminars, which are aimed at deepening the understanding of learning content through interactive exchange. Often, students' cameras and microphones remain switched off, so that communication is limited to chat [1], [2], [3]. This means that, in contrast to an in-person seminar, students' facial expressions, gestures, voice pitch, and appearance are missing. This lack of verbal and nonverbal signals can disrupt *social presence* [4], i.e., the subjective impression of being in the company of a real person who is accessible and responsive in the conversational setting [5], [6]. Low levels of social presence in videoconferences reduce student satisfaction and participation in the form of questions and comments [7], [8], [9]. In a case study by Massner, lecturers complained that due to this lack of student feedback and their own gestural limitations they could not teach the same way in videoconferences as they were used to doing during in-person seminars [9]. 

The limited interaction in videoconferencing can also be attributed to the novel phenomenon of *Zoom fatigue*, which has been increasingly reported since the beginning of the COVID-19 pandemic. Zoom fatigue is a fatigue syndrome believed to be triggered by the heavy use of videoconferencing, and is not limited to its eponymous provider, Zoom [10]. When students suffer from Zoom fatigue, they find it harder to concentrate and participate during the online seminar [3], [9]. Various causes are being discussed for this phenomenon. As with a lack of social presence, one possible reason is limited verbal and nonverbal communication [10], [11], [12]. Even when the participant's video camera is turned on, video conferencing lacks gestures, positioning in space, and the option to detect subtle changes in facial and vocal expressions. Combined with latency in video and audio transmission, this lack of signals makes it difficult to interpret contributions and anticipate pauses in speech [9], [10]. This is compounded by the peculiar view in video conferencing. The grid view in video streams gives the impression of being under constant surveillance and tempts people to check their own video image regularly. These adverse conditions in videoconferencing impede the two-way flow of conversation and exhaust the limited working memory [9], [10], [13], which plays a central role in the processing and long-term storage of information.

The workload of the working memory – known as *cognitive load* – is an important measure of the suitability of a teaching method and is differentiated into three cognitive processes [14]. While the *intrinsic load* is determined by the complexity and novelty of the learning content, the *extraneous load* describes the extent to which the working memory is taxed by the instructional design (e.g., the grid view in videoconferences). This load should be low to leave as much working memory capacity as possible available for the actual learning process in the form of *germane load*. Video-based online seminars are suspected of causing a high extraneous load and thus promoting Zoom fatigue [9], [10]. While this does not necessarily mean that videoconferencing leads to reduced learning success compared to in-person seminars [15], it does inhibit student interaction, participation, attention, and satisfaction [9], [15], [16]. Along with social presence and cognitive load, these factors are used to describe the learning experience of students in the present field study.

#### 1.2. Conferencing in virtual reality

**Figure 1:**
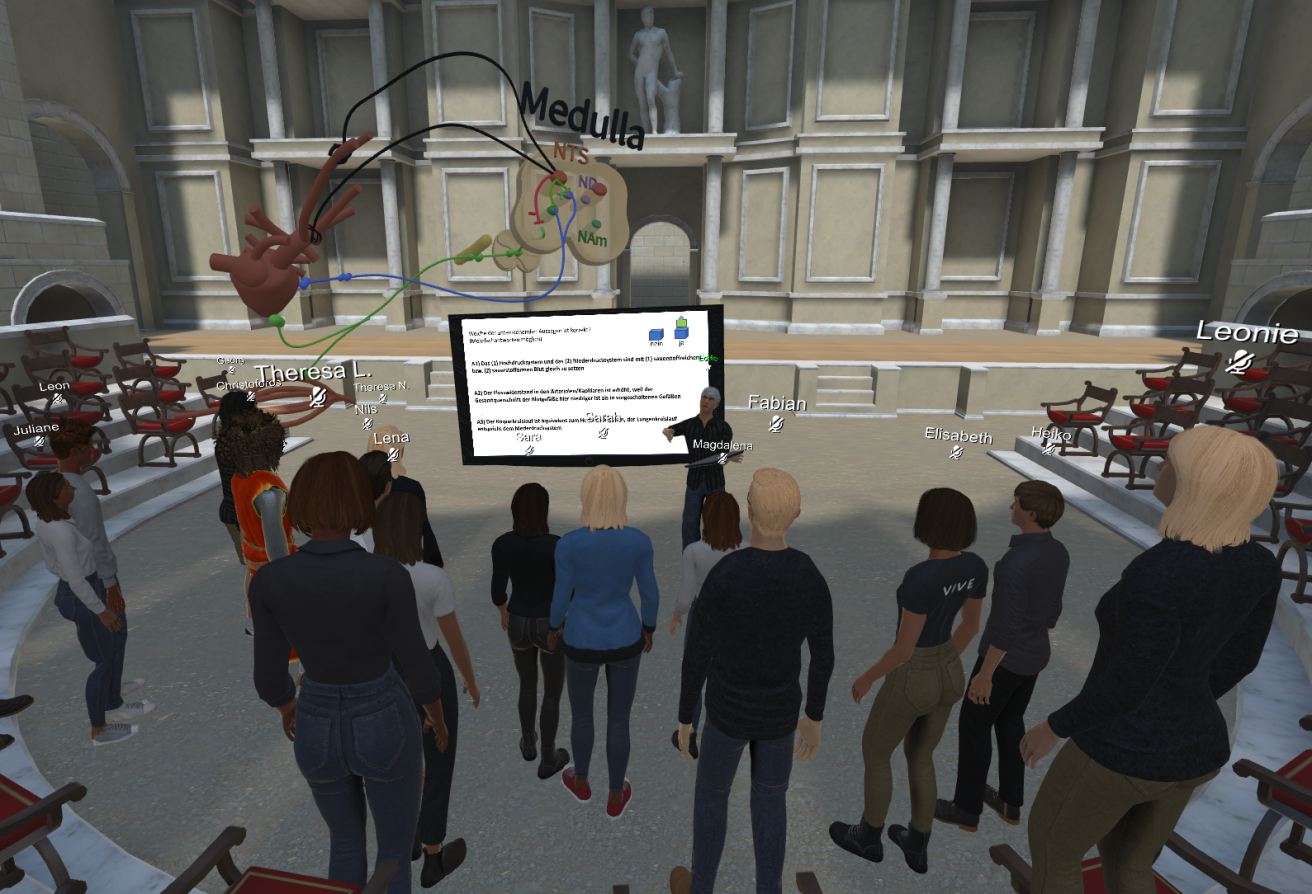
Screenshot of a VR conference. The screenshot above shows a seminar group in a VR conference. The lecturer was using an HMD to teach and illustrated physiological control circuits using 3D models. The students participated via non-immersive devices (PC, laptop or tablet).

One alternative to videoconferencing is conferencing in virtual reality (VR). In VR conferences, lecturers and students meet as avatars in virtual 3D environments, where they can approach each other and interact spatially (see figure 1 (Fig. 1)). While the technological forerunners such as Second Life were still limited to *non-immersive* devices (PC, Mac, laptop, tablet or Smartphone), modern VR conferencing systems such as Mozilla Hubs and Engage also allow for *immersive* participation using head-mounted displays (HMDs). In the latter case, also known as social VR, real gestures and – depending on the equipment – facial expressions are projected into the virtual space. Communication via HMD thus resembles a real conversation and could enable lecturers to teach as if face-to-face using gestures and visible conversation partners, despite the virtual distance. However, this assumption has been poorly substantiated thus far, as teaching experience as an umbrella term for teaching-related behavior (e.g., addressing students) and experience (e.g., perception of student participation) is only mentioned casually in relevant publications [17], [18]. Another unique feature of VR conferencing is the virtual 3D space, which is not bound by natural laws and thus opens up new didactic possibilities. Media (e.g., slides, videos, and 3D models) can be positioned, scaled, and annotated freely within the space by both lecturers and students. For example, students can explore anatomical structures and chemical elements spatially while the lecturer annotates them three-dimensionally.

Unlike lecturers' teaching experience, some study results are already available with regard to the learning experience of students in VR. These, however, do not exhibit consistent findings. For example, the comparison of learning performance between non-immersive and immersive VR varies depending on the study and the application scenario [19], [20], [21], [22], [23]. However, there are several indications that immersion in VR via HMD can increase not only social presence but also extraneous load [17], [19], [20]. So far, this downside of immersive VR has yet to have much impact on current practice, as most students do not own HMDs and thus control their avatars via traditional devices. This in turn reduces their range of nonverbal expressions to automatically synchronized lip movements, positioning in space, and buttons to trigger predefined actions (e.g., raise hand) and reactions (e.g., laugh). This non-immersive participation is viewed in a predominantly positive light [24]. The positive aspects include a high degree of interactivity and fun in the classroom. Non-immersive VR conferencing was also found to offer higher levels of social presence and motivation compared to videoconferencing [25]. In a study by Yoshimura and Borst, some students also noted that they preferred communicating via an avatar to using a webcam [17]. 

#### 1.3. Research questions

In this field study, online seminars in the form of VR conference, videoconference, and independent study were offered, conducted, compared, and – with regard to the teaching experience in VR – contrasted with in-person teaching. The following research questions were investigated:

1. *Lecturers’ teaching experience*
  Does the teaching behavior and experience when using an HMD differ from in-person teaching?
  What are the didactic added values and obstacles associated with VR conferencing compared to videoconferencing?
2. *Choice of teaching format*
  Which teaching format do medical students prefer?
3. *Students' learning experience*
  Which teaching format offers the best learning experience (including social presence and participation)?
4. *Students' learning performance*
  Which teaching format results in the best learning performance (declarative and spatial)?

## 2. Method

### 2.1. Study design 

The study was conducted in two consecutive compulsory online seminars in the third (WS 2020/21; February 2021) and fourth semesters (SS 2021; April and May 2021) of Human Medicine at the Faculty of Medicine at the University of Ulm with approval of the ethics committee of the University of Ulm. The seminars were part of the General Physiology curriculum and focused on physiological control circuits and blood pressure regulation. At the beginning of both seminars, students (*N_WS_*=328; *N_SS_*=308) were able to choose the teaching format, selecting from synchronous seminar participation via (a) VR conferencing or (b) videoconferencing and (c) asynchronous independent study.

Depending on their chosen formats, students completed either two conference sessions of 90 minutes each with an average of 18.73 participants (*SD*=1.44) or two independent study units on the university learning platform Moodle. The independent study units included explanatory videos with 2D graphics on physiological processes and MC questions to ensure understanding. Learning materials were designed by the responsible teaching supervisor and co-author of the study (EF) and defined as content guidelines for the synchronous teaching formats.

Due to the high number of students who opted for videoconferencing, the format was offered by a total of eight different lecturers, including experienced teaching staff and student assistants with limited teaching experience. In the videoconferences, the same content was taught and the same MC questions were asked as in the independent study materials. However, the lecturers retained creative freedom in preparing the specified materials, meaning that the presentation slides differed slightly. By contrast, the only lecturer for the VR conferences, which were identical in content too, was the teaching supervisor, who at the time had seven years of teaching experience and advanced skills in using non-immersive devices (e.g., 3D modeling on a desktop). The VR conferences also included a mandatory technical introduction in VR and 3D models instead of 2D graphics. Students were able to explore the 3D models spatially without manipulating them (e.g., rotating, scaling, or annotating).

#### 2.2. VR conference

The online VR seminar took place via the fee-based Engage conferencing system, which requires students to install the software and create an account to participate. Engage was preferred over free open-source alternatives such as Mozilla Hubs because the service provider offered personal support with technical questions and problems to ensure a smooth teaching process. The lecturer in the VR conferences taught using a Meta Quest HMD (6DoF, 1440×1600 per eye, 100° FOV, 72 FPS, no face tracking) and explained physiological control circuits using 3D models that he himself had created in advance on the desktop and in VR. He was supported in the organization and technical preparation of the sessions by the Competence Center eEducation in Medicine Baden-Wuerttemberg, which employs another one of the authors (RS).

#### 2.3. Instruments and statistical analysis

Data collection, in which the students participated voluntarily, took place in the synchronous teaching formats after the last seminar date in each case and was conducted online using a questionnaire plugin for the university learning platform Moodle [26]. Students participating in the asynchronous independent study format were also asked to complete the questionnaire directly after the end of the seminar. However, students who opted for the independent study format were allowed a period of one week to complete the questionnaire, as these students did not finish the seminar at the same time.

In the 2020/21 winter semester, students assessed their learning experience and success only subjectively by completing a questionnaire. The student learning experience was surveyed in terms of social presence, cognitive load, and the constructs listed in table 1 (Tab. 1). Social presence was measured using a German translation of the Multimodal Presence Scale (MPS) (e.g., *“I felt like I was in the presence of another person in the virtual environment.”*) [27], [28], which includes five items with a response scale ranging from 1 (*“I completely agree”*) to 5 (*“I completely disagree”*). Extraneous (e.g., *“The design of the VR conferences was very inconvenient for learning.”*) and germane cognitive load (e.g.,* “My point while attending the VR conferences was to understand everything correctly.”*) were queried using five items based on the work of Klepsch et al. [29]. Answers were based on a response scale from 1 (*“I completely disagree”*) to 7 (*“I completely agree”*). Since the intrinsic load depends on the learning content, which was the same across all teaching formats, it was not analyzed. The response scale used for cognitive load was also applied to the items created by the authors and listed in table 1 (Tab. 1) and the subjective questions on declarative learning content (e.g., *“I have understood why vasoconstriction of resistance vessels leads to an increase in blood pressure.”*) and its spatial location (e.g., *“I can spatially locate individual elements of the models/graphs shown.”*) (see attachment 1 , tables A1 and A2). Social presence and the constructs of interaction and participation were not measured in the independent study group because the instructional format did not include any social interaction.

In order to objectively validate the assessment of learning success, a voluntary single-choice knowledge test was administered in all teaching format groups during the 2021 summer semester. The test included eight questions, each with five answers to choose from (see attachment 1 , table B1). The solutions for the knowledge test, which was advertised as an opportunity for additional exam preparation, were made available to all students in the seminar after the questionnaire was completed. The learning experience was not re-surveyed. Data collected over the two semesters were adjusted and analyzed using SPSS (Version 27) and are presented using the arithmetic mean (*M*), standard deviation (*SD*), and supplemental qualitative student feedback.

Data about the teaching experience of the lecturer who taught in the VR conferences was collected during a semi-structured interview in late February 2021. The interview questions in attachment 1 , table C1, were aimed at contrasting the VR teaching experience and behavior with those during in-person teaching (e.g., *“To what extent did teaching via head-mounted display (HMD) differ from in-person teaching?”*) and identifying possible added values and obstacles of VR conferencing compared to videoconferencing (e.g., *“In your opinion, do VR conferences have any added didactic value compared to video-based online seminars?”*). The interview was recorded and transcribed by hand. The statements collected were filtered based on their relevance to the two research questions on teaching experience, then sorted and summarized as continuous text including direct quotes.

## 3. Results

### 3.1. Sample

Table 2 (Tab. 2) describes the samples differentiated by teaching format and semester. Participation rates increased from 28% (*n_WS_*=93) to 60% (*n_SS_*=158) across groups between semesters. While sex, age, and affinity for technology were similar across groups, the VR variant showed a descriptively higher interest in VR technology.

#### 3.2. Teaching experience

##### 3.2.1. Teaching behavior and experience

Due to the ability to move spatially and gesture freely with the HMD, the lecturer's teaching experience in VR was similar to that during in-person teaching:

*“The classic teaching situation […] is mirrored one-to-one in the VR conference. I was completely immersed in the world. Whether that was a virtual or real wall no longer made a difference to me.”*

At the beginning of the seminar, the students seemed like *“mannequins”* to the lecturer, since their natural gestures and facial expressions could not be reproduced via conventional devices. However, this impression faded into the background in the course of the class. The lecturer estimated that active student participation was higher in VR than in videoconferencing. The generic, non-verbal expressions of the students (e.g., nodding and shaking their heads) helped the lecturer to assess comprehension and attention, but was not equivalent to the real facial expressions and gestures in in-person teaching.

##### 3.2.2. Added values and obstacles

The lecturer stated that the greatest added value of VR was the possibility it afforded to teach almost as in-person despite social distancing requirements. Another advantage was the variety of didactic possibilities. In contrast to videoconferences, media (e.g., slides, videos, and 3D models) and tools (e.g., questionnaires and free drawing in 3D) could be used and modified freely within the virtual space. This meant, for example, that students could spatially explore 3D models such as anatomical structures and chemical elements. However, users should be mindful of scaling the 3D models and lettering in the virtual space sufficiently large for smaller displays like tablets and smartphones. Although the initial effort required to prepare for VR conferences (choice of conference system, technical familiarization with hardware and software, and design of 3D models), represents a significant obstacle, this would be reduced in subsequent seminars, as with other teaching formats. In everyday practice, VR conferences therefore do not necessarily require more work than videoconferences and in-person teaching.

#### 3.3. Choice of teaching format

**Figure 2:**
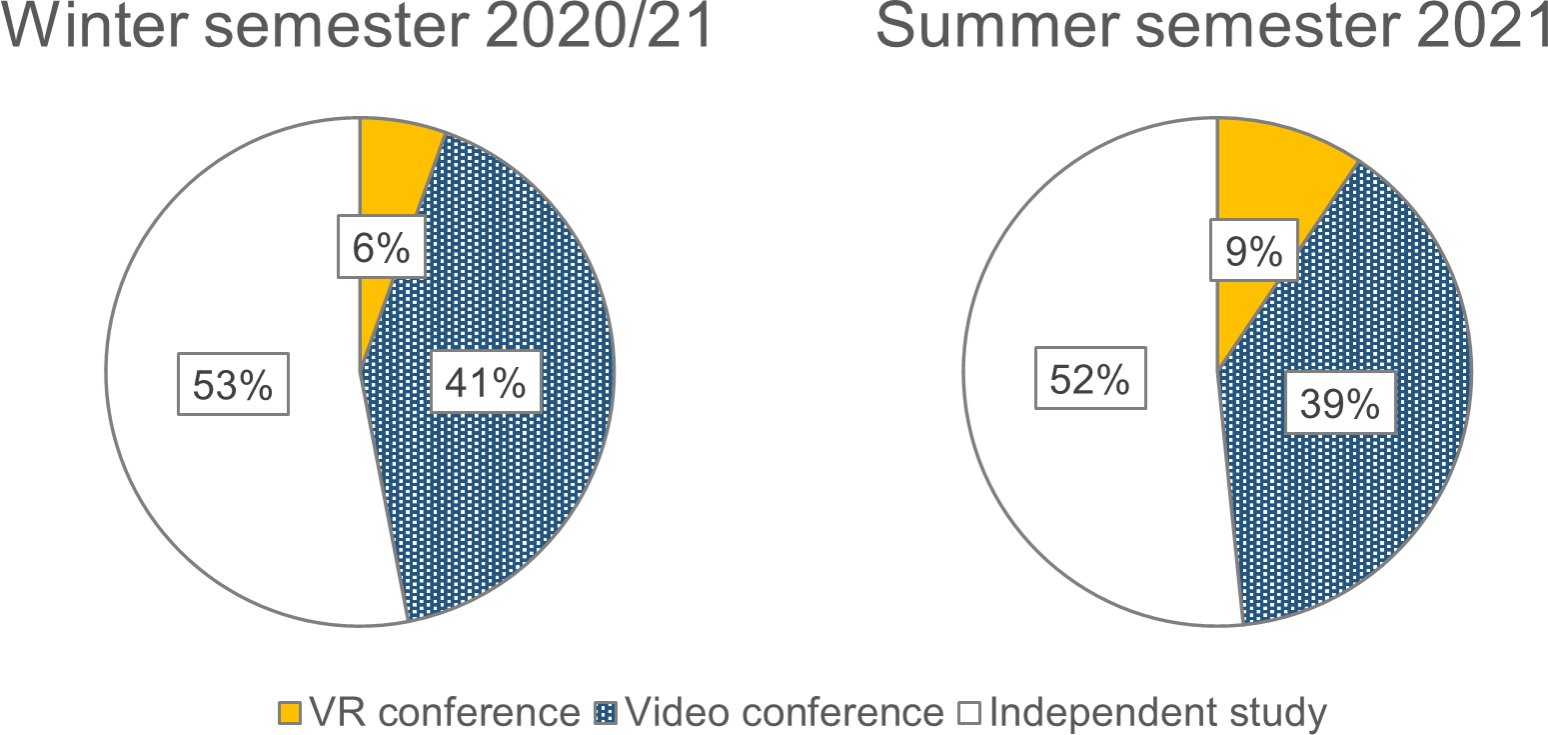
Ratio of chosen teaching formats. The ratio of chosen teaching formats refers to the total number of seminar participants (*N_WS_*=328; *N_SS_*=308).

In both semesters, the majority of students opted for independent study (*N_WS_*=174, *N_SS_*=159). In an optional query, the most frequently cited reasons for this decision were free time management (*n_WS_*=35), a preference for independent study (*n_WS_*=19), and less work (*n_WS_*=8). The second most common choice in both semesters was videoconferencing (*N_WS_*=136, *N_SS_*=120) due to social exchange (*n_WS_*=6) and the opportunity to ask questions (*n_WS_*=4). Significantly fewer students attended the VR conferences (*N_WS_*=18, *N_SS_*=29), but attendance increased by 61% between semesters (see figure 2 (Fig. 2)). The most common motivations to choose VR conferences were curiosity (*n_WS_*=14) and a desire for change (*n_WS_*=3):

*“I felt like trying something new. I haven't had much exposure to VR before, and I've never participated in anything like [the VR conference], so I was very excited to see what the seminar would be like.”*

#### 3.4. Learning experience

**Figure 3:**
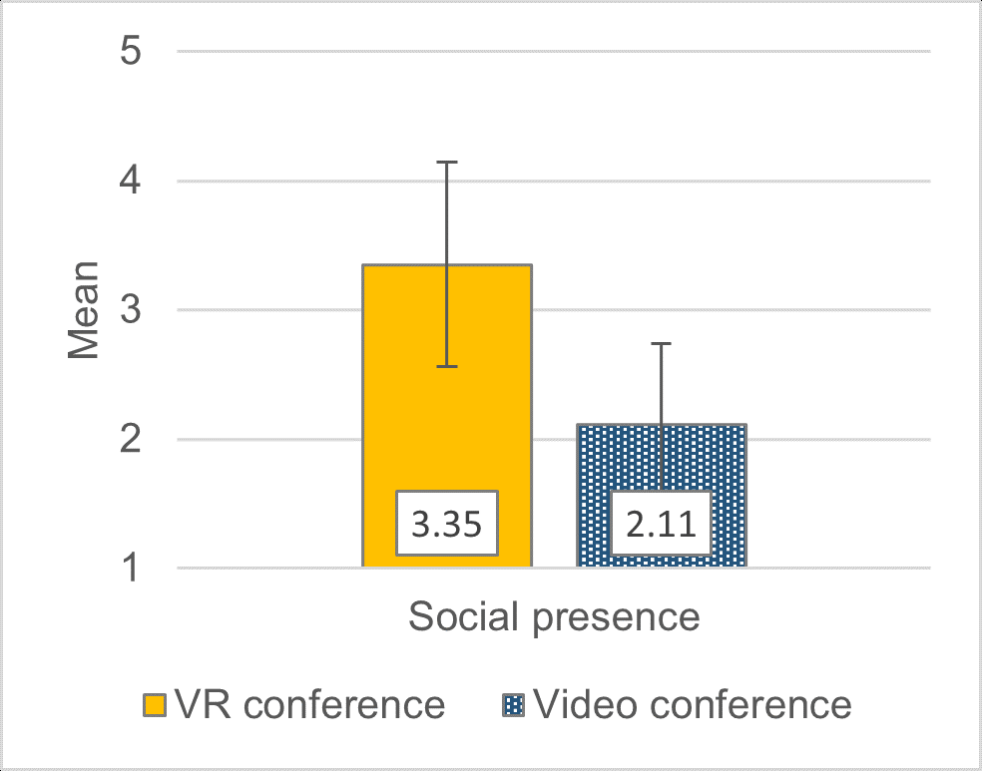
Sense of social presence in conference formats. Multimodal Presence Scale (MPS) by Makransky et al. [19] with a response scale of 1 ("I completely disagree.") to 5 ("I completely agree.") [27], [28]. +/- 1 SD error bars.

The average extraneous load in the VR conference group was lower (*M*=2.14, *SD*=1.04) than that in the videoconferences (*M*=3.23, *SD*=1.08) and during independent study (*M*=3.12, *SD*=1.28). However, the latter performed better with regard to germane load (*M*=5.54, *SD*=1.02) than the VR conference (*M*=4.91, *SD*=1.29) and the videoconference groups (*M*=4.75, *SD*=1.68). In contrast to videoconferences, the VR conference format also induced a moderate sense of social presence (see figure 3 (Fig. 3)).

**Figure 4:**
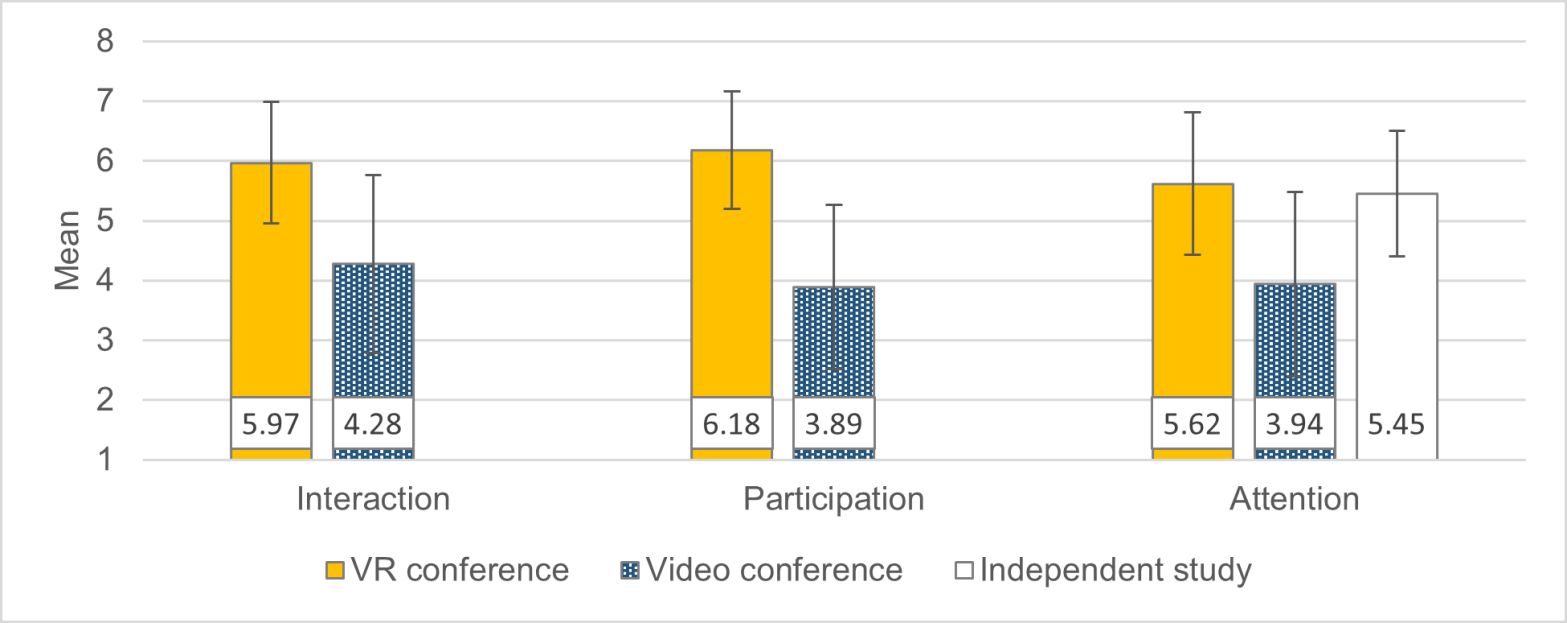
Interaction, participation, and attention ratings. The items for the three factors shown are listed in table 2. Response scale from 1 (“I completely disagree.”) to 7 (“I completely agree.”). +/- 1 SD error bars.

Students considered VR conferencing more suitable for interaction in seminars than videoconferencing. Subjective attention and participation were also rated higher in VR than in videoconferencing (see figure 4 (Fig. 4)):

*“Personally, I noticed that I stay on task much better and listen better in the VR condition. In [videoconferences] you often get distracted quickly or do something else on the side. That was not the case [in the VR conference].”*

The winter semester seminar was rated higher with regard to both German school grades (SG), ranging from 1 (*“very good”*) to 6 (*“unsatisfactory”*), and experienced enjoyment (E) in the VR conference group (*M_SG_*=1.29, *SD_SG_*=.47; *M_E_*=6.76, *SD_E_*=.56) than in the independent study (*M_SG_*=2.12, *SD_SG_*=.61; *M_E_*=5.02, *SD_E_*=1.30) and videoconference groups (*M_SG_*=2.38, *SD_SG_*=.89; *M_E_*=4.44, *SD_E_*=1.37):

*“For me personally, [VR conferences] are the best online-based option for seminars that cannot be held in person due to the various illustrative models and the possibility of direct interaction in the “lecture hall”.”*

#### 3.5. Learning performance

**Figure 5:**
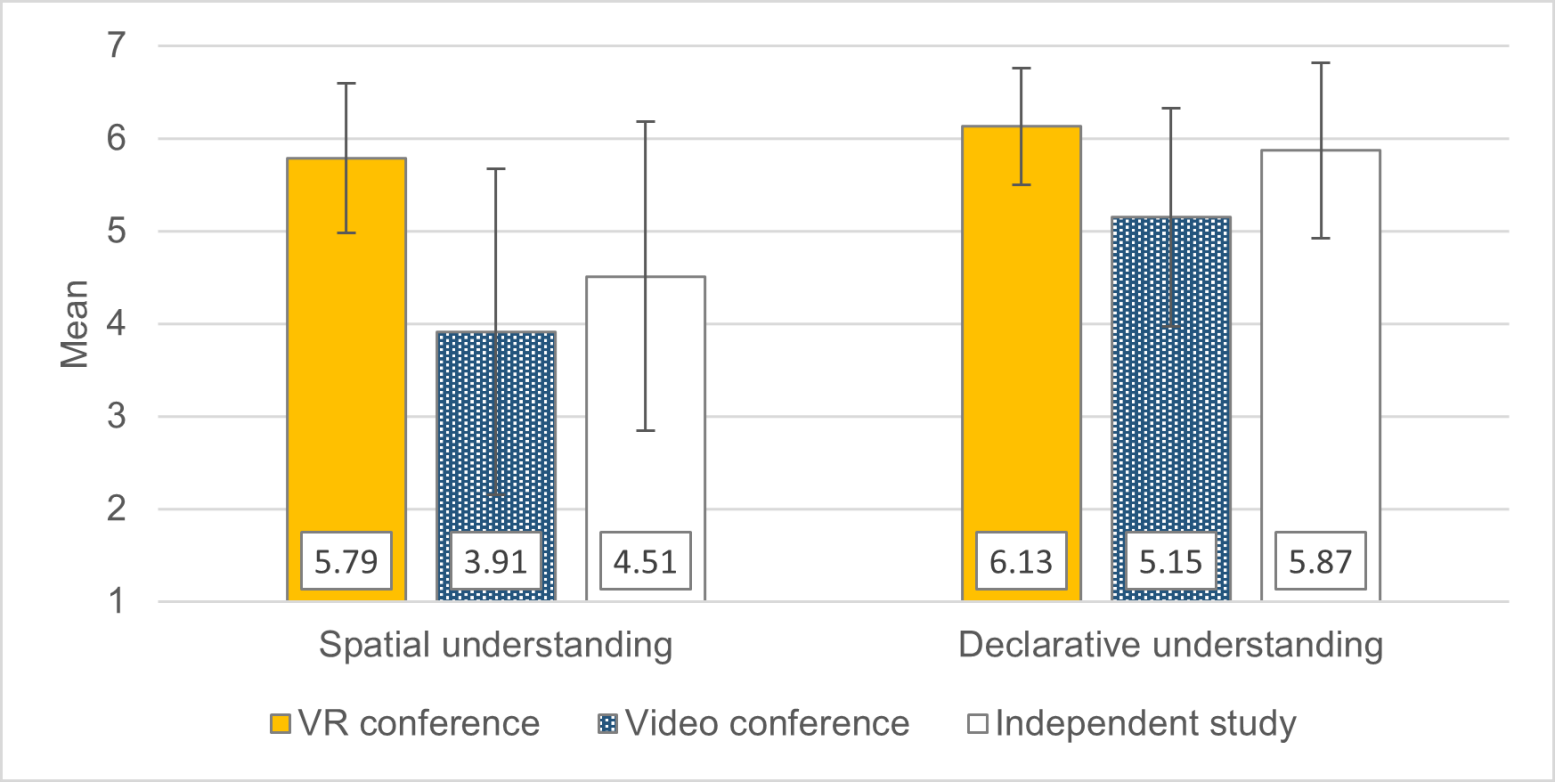
Subjective learning performance in the 2020/21 winter semester. The items for the two factors shown are listed in attachment 1. Response scale of 1 (“I completely disagree.”) to 7 (“I completely agree.”). +/- 1 SD error bars.

**Figure 6:**
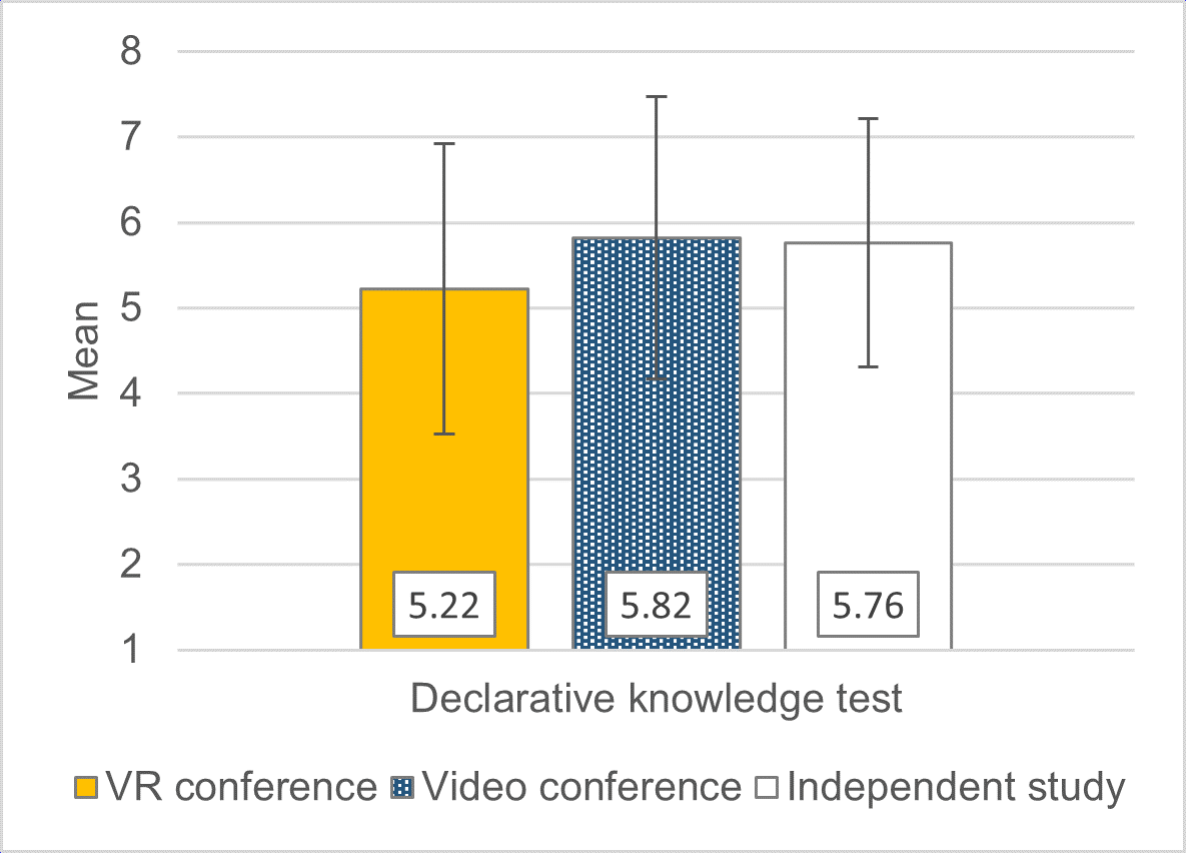
Objective learning performance in the 2021 summer semester. The knowledge test consisted of eight questions, each worth one point. The questions are listed in attachment 1. +/- 1 SD error bars.

Spatial comprehension was better in the VR conference group than in the other two formats. With regard to declarative comprehension, the preference for VR is descriptively only found when compared to videoconferencing (see figure 5 (Fig. 5)). In the objective knowledge test, where no format stood out, the VR conference group performed slightly worse descriptively (see figure 6 (Fig. 6)).

## 4. Discussion

VR conferencing is an alternative to videoconferencing, which has been associated with Zoom fatigue and low student participation, among other issues [1], [2], [30], [31]. In this field study, the two conference variants and the independent study format were compared in curricular use and – in the case of teaching experience in VR – contrasted with in-person teaching. 

For the lecturer in VR, the initial preparation of the VR conference was a large time investment, despite prior knowledge and support. Therefore, it can be assumed that HMDs are currently only used for teaching by lecturers interested in the technology who can afford the preparation time. This assumption is reinforced by thematically-related project reports [17], [18]. If VR is to reach a broader range of faculty, the barrier to entry must be reduced through faculty support services including the pre-selection and provision of HMDs and VR conferencing systems for which technical and didactic training is provided. Such offerings can enhance the teaching experience, especially during the pandemic and in international degree programs for which in-person teaching is not possible. Instead of lecturing in front of a webcam, lecturers can use HMDs to teach interactively and motivate their students almost as if they were in a real seminar room. VR also opens up new didactic possibilities such as spatial teaching and learning with 3D models. The latter can be downloaded ready-made from platforms such as Sketchfab or drawn in 3D by the lecturer using intuitive VR software (e.g., gravity sketch). 

Students preferred the established teaching formats in their choice of format. The reasons given for choosing independent study were free time management and a preference for independent learning, which synchronous teaching cannot offer. With regard to the synchronous teaching formats, only a minority chose VR conferencing, which can be explained by the varying interest in VR technology and the additional work involved (written instructions, software installation, and test conferencing) according to the Technology Acceptance Model [32], [33]. VR conferencing will probably only become attractive to the broader student body with increasing awareness and improved accessibility (use without installation and account creation) [34], [35]. If VR conferencing is mandatory, technical instruction should be provided in advance, otherwise learning success will be determined by individual technical competence [25], [36]. 

The students who opted for the VR variant despite the extra work it entailed did so mostly out of curiosity and rated the seminar and their learning experience as better overall than their fellow students. The increase in the number of participants between surveys is thus possibly due to students recommending the VR variant. The high scores for motivation, attention, and interaction are consistent with previous research on non-immersive VR conferencing [24], [25], [37], [38], [39], [40], [41], [42], [43]. By contrast, the results on social presence were unexpected in relation to videoconferencing. According to Bailenson et al. (2018), the feeling of being in virtual company should not differ noticeably between videoconferencing and VR conferencing [44]. The difference found may be a result of the Zoom fatigue that currently prevails and the enthusiasm of the VR group. Interaction, attention, and participation were also higher in the VR conferences. One reason for the high level of activity in these sessions seems to be the use of virtual avatars. In contrast to videoconferences, students in VR seminars are always physically represented and recognizable as counterparts without being visible in real life [45], [46]. Thus, students have no reason to monitor their actual appearance during the VR conference, which possibly accounts for the lower extraneous load. Finally, students also rated their own spatial and declarative learning performance highest in the VR group. As this tendency was not objectively confirmed with respect to the declarative content, however, the reliability of the student data must be questioned in a critical light. Nevertheless, the evaluation of spatial understanding appears to be credible, since it is only possible to explore visual material three-dimensionally in VR conferencing.

In contrast to VR conferencing, videoconferencing performed worst in all subjective aspects of the learning experience. This includes an unfavorable working memory load, which has already been identified by other authors as a possible cause of Zoom fatigue [10], [11], [12]. This is noteworthy in light of the fact that the format was selected by the students themselves and suggests that videoconferencing is also frequently perceived by students as fatiguing and ineffective. The wide variation in responses suggests that the quality and suitability of videoconferences is dependent on individual preferences and teaching styles. The declarative knowledge test also gave the authors no reason to believe that videoconferencing impairs declarative learning success.

When interpreting the results, it must be taken into account that the students themselves chose their groups based on their personal preferences and that different lecturers taught in the various conferences. In addition, the ratings could have been influenced by the novelty effect [47]. Accordingly, the positive evaluation of VR conferences on the part of the lecturer and the students could be relativized once VR technology has become established. In order to determine whether the tendencies identified in this study are generally valid, they need to be tested with randomized groups in more controlled settings. Future studies should also investigate how participation type (immersive vs. non-immersive) and upcoming technological innovations (e.g., photorealistic avatars with authentic facial expressions) affect VR conferences in teaching [11]. 

## 5. Conclusions

The findings from curricular teaching show that VR conferencing can enrich synchronous distance teaching if lecturers and students are open to the technology. VR conferencing offers lecturers who invest the initial effort new didactic opportunities in a virtual 3D space and a teaching experience similar to in-person teaching. Most students prefer videoconferences and independent study, which require less preparation. However, interaction, participation, social presence, and the seminar, among other things, are rated higher in VR conferencing than in established teaching formats. Nevertheless, this subjective assessment does not result in better declarative learning performance.

## Funding

The field study was financially supported by the AG Lehrforschung (Committee on Medical Educational Research) of the Faculty of Medicine at the University of Ulm. 

## Competing interests

The authors declare that they have no competing interests.

## Supplementary Material

Additional tables

## Figures and Tables

**Table 1:**
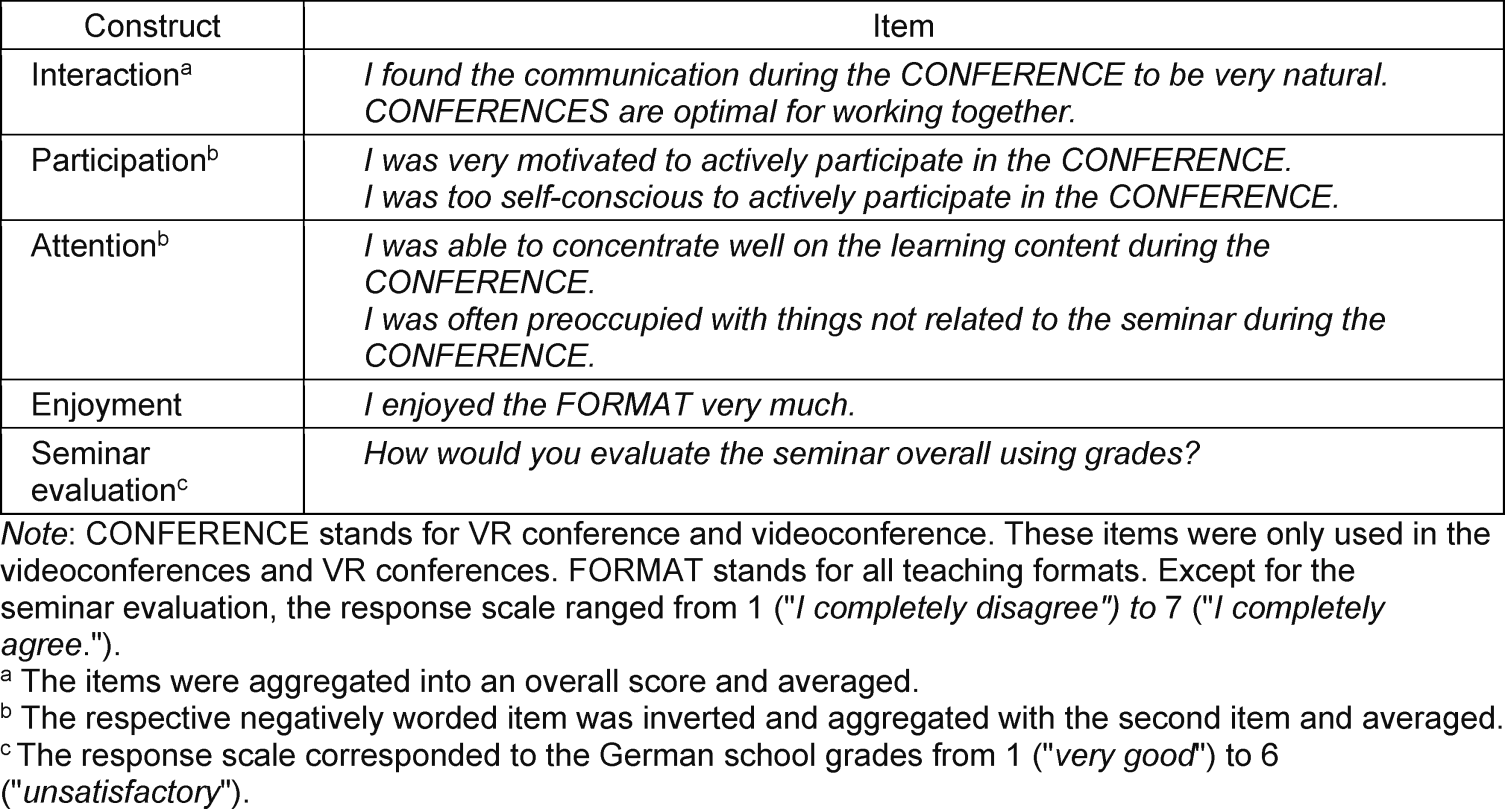
Questionnaire items created by the authors and used in winter semester 2020/21

**Table 2:**
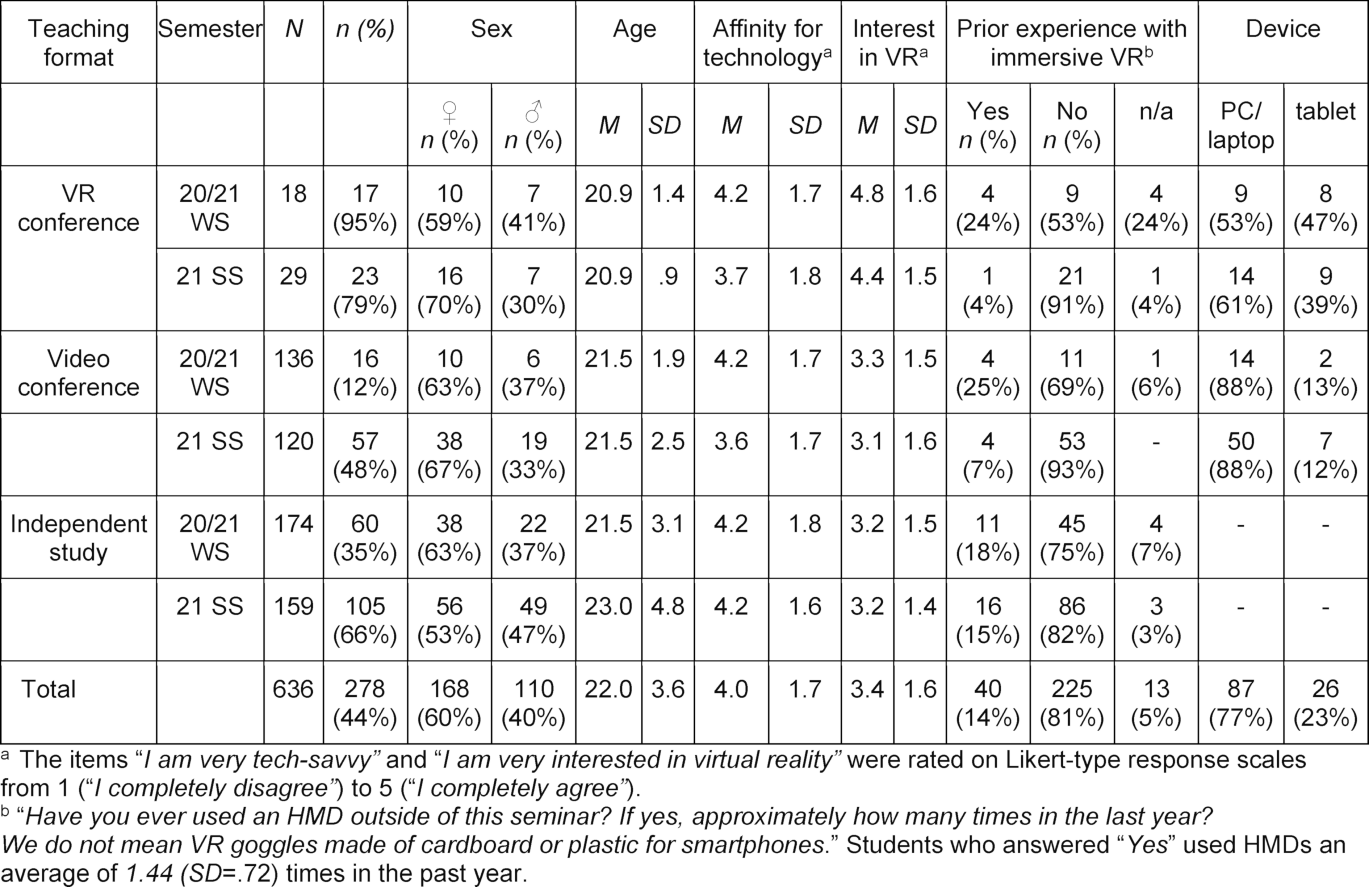
Sample description
