# Supplementary material for: Virtual reality against Zoom fatigue? A field study on the teaching and learning experience in interactive video and VR conferencing
Source: GMS J Med Educ. 2023 Apr 17;40(2):Doc19. doi: 10.3205/zma001601 (PMC10285369; doi:10.3205/zma001601)
Supplement: Additional tables [file JME-40-19-s-001.pdf]

Attachment 1: Additional tables

Table A1: Declarative understanding

ID	Item
1_factual	I have understood which "sensors" play a role in SBR.
2_factual	I have understood why vasoconstriction of resistance vessels leads to an increase in blood pressure.
3_factual	I have understood the principal mechanisms of action of sympathetic/parasympathetic innervation on the effectors.
4_factual	I have understood the principal differences between SBR and LBR.
5_factual	I have understood which sub-aspects of LBR were excluded from the seminar and the role played in LBR by the renin-angiotension-aldosterone system, which was discussed in more detail during the session.
6_factual	I have understood the conditions under which renin release occurs and how this affects sodium uptake in the collecting duct.

Note. Short-term (SBR) and long-term blood pressure regulation (LBR). The items were aggregated into one value and averaged. The response scale ranged from 1 ("I completely disagree") to 7 ("I completely agree.").

Table A2: Spatial understanding

ID	Item
1_spatial	I can still visualize the models/graphics shown in detail.
2_spatial	I can spatially locate individual elements of the models/graphics shown.

Note. Items were aggregated into one value and averaged. The response scale ranged from 1 ("I completely disagree.") to 7 ("I completely agree").

Table B1: *Questions and responses from the declarative knowledge test*

ID	Item	Response options
		Topic "Ventilation"
1_test	Which definition of "vital capacity" is correct?	**Volume that can be inhaled after maximum expiration when breathing in as deeply as possible.**
		Volume that can be exhaled within one second during forced expiration.
		Volume that is inhaled or exhaled during normal breathing.
		Volume that is in the airways that do not participate in gas exchange.
		Volume that remains in the lungs after maximum expiration in a patient with obstructive lung disease.
2_test	Which statement is true if airway resistance is increased?	The one-second capacity (FEV1) is increased.
		The respiratory flow is increased.
		**The curve in the flow-volume loop is compressed on the Y-axis.**
		The vital capacity is reduced.
		The residual volume is reduced.
3_test	Which statement about intrapleural pressure is true?	It is always negative.
		It cannot be lower than the pulmonary pressure.
		It corresponds to the atmospheric pressure in a "closed system" in the resting expiratory position.
		**It corresponds to the atmospheric pressure in a "closed system" when the respiratory muscles are relaxed and the ribcage is in its resting position.**
		It corresponds to the atmospheric pressure in an "open system" when the ribcage is in its resting position.
4_test	Which statement about the acid-base balance is true?	Standard bicarbonate typically changes in the event of a disturbance in the respiratory acid-base balance.
		Hyperventilation causes respiratory acidosis.
		Hypoventilation causes metabolic acidosis.
		If lactate is produced during physical activity, the body can exhale it through hyperventilation.
		**Metabolic acidosis can be offset by hyperventilation.**
		Topic "Circulation"
5_test	Which statement about the high-pressure system is correct?	An increase in peripheral resistance can be expected to decrease blood pressure.
		The arterial section of the pulmonary circulation system is part of the high-pressure system.
		If cardiac output increases, the stroke volume of the heart must also increase.
		The Hagen–Poiseuille law describes the diffusion properties of a gas.
		**Cardiac output and peripheral resistance are the decisive variables in short-term blood pressure regulation.**

Attachment 1 to Speidel R, Felder E, Schneider A, Öchsner W. *Virtual reality against Zoom fatigue? A field study on the teaching and learning experience in interactive video and VR conferencing.* GMS J Med Educ. 2023;40(2):Doc19. DOI: 10.3205/zma001601

6_test	Which statement about flow and pressure pulse is correct?	Both can only be determined in central vessels (e.g., the aorta).
		The propagation speed of the pressure pulse is slower than the flow velocity of the blood.
		The flow pulse is higher in the periphery than in central vessels.
		**The pressure pulse may be higher in the periphery than in central vessels.**
		The pressure pulse is determined using Doppler ultrasound.
7_test	What happens when you stand up (orthostasis)?	The muscle pump in the legs begins to pump blood to the heart.
		**The blood in the venous system sinks into the legs.**
		The Frank–Starling mechanism causes more blood to be pumped into the high-pressure system.
		The stroke volume of the heart increases acutely.
		The heart rate decreases.
8_test	Which statement about performance physiology is correct?	**The 9-panel plot diagram compares ventilation parameters, concentration of respiratory gases, and pulse rate.**
		The end-tidal CO2 concentration is determined at the end of inspiration.
		Acidosis caused by physical exertion is combated by hypoventilation.
		If physical exertion is too great, the pH may rise to the point where the subject must stop the activity.
		Respiratory minute volume is the volume of oxygen consumed in one minute.

Note. Correct answers are marked in bold and were aggregated into a total score for analysis.

Table C1: Qualitative interview questions for the VR conference teaching format

Research question	Questions
Teaching behavior and experience	To what extent did teaching via head-mounted display (HMD) differ from in-person teaching?
	Were you able to assess whether students were following your teaching during the VR conference?
	Did students participate in VR to the extent you would expect in one of your in-person seminars?
Added value and obstacles	In your opinion, do VR conferences have any added didactic value compared to video-based online seminars? If yes, what is it?
	Where do you see obstacles to establishing VR conferences in teaching?
	How do you estimate the learning success of students in VR conferences compared to those in video-based online seminars?
	How much work do you estimate VR conferencing takes compared to video-based online seminars?
	Is IT affinity or expertise a prerequisite for teaching through VR conferences?
